# Hope amidst neglect: Mycetoma Research Center, University of Khartoum. A holistic management approach to achieve the United Nations’ Sustainable Development Goals

**DOI:** 10.1371/journal.pntd.0012420

**Published:** 2024-09-05

**Authors:** Ahmed Hassan Fahal, Iman Siddig Ahmed, Ali Awadallah Saaed, Dallas J. Smith, Fabiana Alves, Borna Nyaoke, Kingsley Asiedu, Roderick Hay

**Affiliations:** 1 The Mycetoma Research Center, University of Khartoum, Khartoum, Sudan; 2 The National University, Khartoum, Sudan; 3 Mycotic Diseases Branch, U.S. Centers for Disease Control and Prevention, Atlanta, Georgia, United States of America; 4 Drugs for Neglected Diseases Initiative, Geneva, Switzerland; 5 Drugs for Neglected Diseases Initiative, Nairobi, Kenya; 6 World Health Organization, Geneva, Switzerland; 7 King’s College, London, United Kingdom; Albert Einstein College of Medicine, UNITED STATES OF AMERICA

## Abstract

Mycetoma is a debilitating neglected tropical disease that affects individuals worldwide, particularly in regions where there is poverty and limited health care access. The Mycetoma Research Center (MRC), based in Khartoum, Sudan, provides a sustainable, holistic approach to patient care as the only World Health Organization collaborating center for mycetoma. We describe MRC activities that align with the United Nations’ Sustainable Development Goals to control mycetoma in Sudan and globally.

## Introduction

Mycetoma is a debilitating condition that affects individuals worldwide, particularly in regions where poverty and limited access to healthcare prevail [1,2]. The Mycetoma Research Center (MRC), based in Sudan at the University of Khartoum, is a critical force in combating this disease, providing not only medical solutions but also, a holistic approach to caring for patients with mycetoma [3].

The MRC is a center of scientific inquiry, patient care, and collaborative efforts. Its primary mission revolves around the development of effective diagnostics and treatments for mycetoma, aiming to alleviate the suffering of patients and prevent the spread of the disease. Through evidenced-based research conducted by a specialist multidisciplinary team, the MRC pioneers innovative approaches pushing the boundaries of medical knowledge and practice [4].

The impact of the MRC transcends the confines of mycetoma research. Recognizing the interconnected nature of global health challenges, the center actively engages in initiatives that resonate with the United Nations Sustainable Development Goals (SDGs) [5]. By aligning its activities with these broader aspirations, the MRC exemplifies a holistic approach to well-being, addressing not only the immediate health concerns but also the underlying social determinants of health.

## Mycetoma

Mycetoma causes devastating morbidity and degrades quality of life. This inflammatory disease is caused by one of more than 70 fungi or bacteria [6]. Mycetoma initially presents with small subcutaneous masses that gradually extend, infiltrating the skin and deeper tissues, including the bone. Its progression is not merely physical, with massive tissue destruction, but it also leaves behind a trail of disability and stigma [6].

Mycetoma shows no respect for age, targeting both young adults and children. Its impact is most acutely felt in the marginalized tropical and subtropical regions, although it is reported globally, where poverty and lack of access to health facilities prevent proper care [7,8]. In these impoverished rural areas, the presence of mycetoma is not just a medical concern but a societal blight, perpetuating cycles of suffering and hardship [9,10].

Mycetoma casts a long shadow with socioeconomic ramifications, weighing heavily on the shoulders of already burdened communities. Disability becomes a harsh reality, restricting mobility and productivity [11]. Social stigma compounds hardship, isolating individuals and their families from the support networks they desperately need [11,12].

## The Mycetoma Research Center

Established in 1991, the Mycetoma Research Center at the University of Khartoum is an inspiration in the fight against mycetoma and other skin neglected tropical diseases (NTDs). Recognized by the World Health Organization (WHO) as the only collaborating center on mycetoma and skin NTDs, the MRC assumes a critical role in the global effort to combat these debilitating afflictions [4].

With a mandate firmly rooted in compassion and scientific excellence, the MRC is dedicated to offering affected patients the most effective evidence-based treatments and management strategies available [13–17]. Its team of dedicated healthcare professionals works to alleviate the suffering of those afflicted by mycetoma, providing not just medical care but also a sense of hope and dignity in the face of adversity. As of 2024, over 12,000 patients with mycetoma have been cared for through the Mycetoma Research Center.

Beyond its clinical endeavors, the MRC serves as a hub for cutting-edge scientific research, pushing the boundaries of knowledge in the field of mycetoma. Through rigorous investigations and innovative approaches, the center seeks to unravel the mysteries surrounding the disease, paving the way for novel diagnostic and treatment modalities and preventive measures. The center conducted the first ever comparative clinical trial on a new drug for mycetoma and has designed many molecular diagnostic tests for the disease [18,19].

Comprehensive training programmes and educational initiatives lie at the heart of the MRC’s mission, as they empower healthcare professionals with the knowledge and skills needed to combat mycetoma effectively both within Sudan and beyond its borders [4].

Community engagement forms the cornerstone of the MRC’s approach as it endeavors to foster meaningful partnerships with affected communities. By actively involving community members in decision-making processes and raising awareness about mycetoma, the center seeks to combat stigma, promote early detection, and facilitate access to care for all those in need, e.g., through outreach programmes [12].

The MRC has earned global recognition as a world authority in the field of mycetoma. Its contributions extend beyond the confines of Khartoum, touching the lives of countless individuals around the world who have been affected by this devastating disease (Table 1). As the MRC continues to forge ahead in its mission, it remains a center of excellence in the control of mycetoma.

**Table 1:** Quantitative impact of the Mycetoma Research Center on Mycetoma during 1991–2024.

Indicator	Impact
Number of patients served overall	>12,000
Number of satellite centers	2
Number of patients served at satellite centers	>5,000
Number of patients with mycetoma served at satellite centers	>1,000
Trained health care providers in endemic regions	>700
Number of villages surveyed for mycetoma	>300
Number of prostheses	200
Number of shoes distributed	800
New animal enclosures	72
Trained patients at the Mycetoma Vocational and Entrepreneurs Center	250
International Collaborating partners	25
National Collaborating partners	15
Number of publications	>300
Number of post-graduate students	30
Number of social media, communication products, printed material	>250
Number of organized international conferences and seminars	6
Presentation on mycetoma at different international meetings	200

## The United Nations Sustainable Development Goals

Achieving the SDGs is imperative for humanity’s collective well-being, offering a blueprint for tangible progress towards ending poverty, hunger, and inequality while promoting education, gender equality, clean water, sustainable cities, climate action, and more [5]. Central to the SDGs is the principle of leaving no one behind, addressing interconnected challenges like poverty, environmental degradation, and lack of essential services to ensure every individual has the opportunity to thrive. Economic prosperity is intertwined with SDG attainment, as eradicating poverty and fostering inclusive growth lay the foundation for sustainable development. Additionally, prioritizing social justice and equity, including gender equality and access to education and healthcare, creates a more just and inclusive world. Environmental sustainability is vital in mitigating existential threats of climate change, with the SDGs advocating for sustainable consumption, conservation of resources, and biodiversity protection. Ultimately, achieving the SDGs is about creating a better future for all, grounded in sustainability, equity, and social justice. To realize this shared vision of prosperity and equity for future generations, the time for action is now.

## Mycetoma Management: The MRC Strategic Blueprint for Achieving the UN’s Sustainable Development Goals by 2030

**Fig 1:**
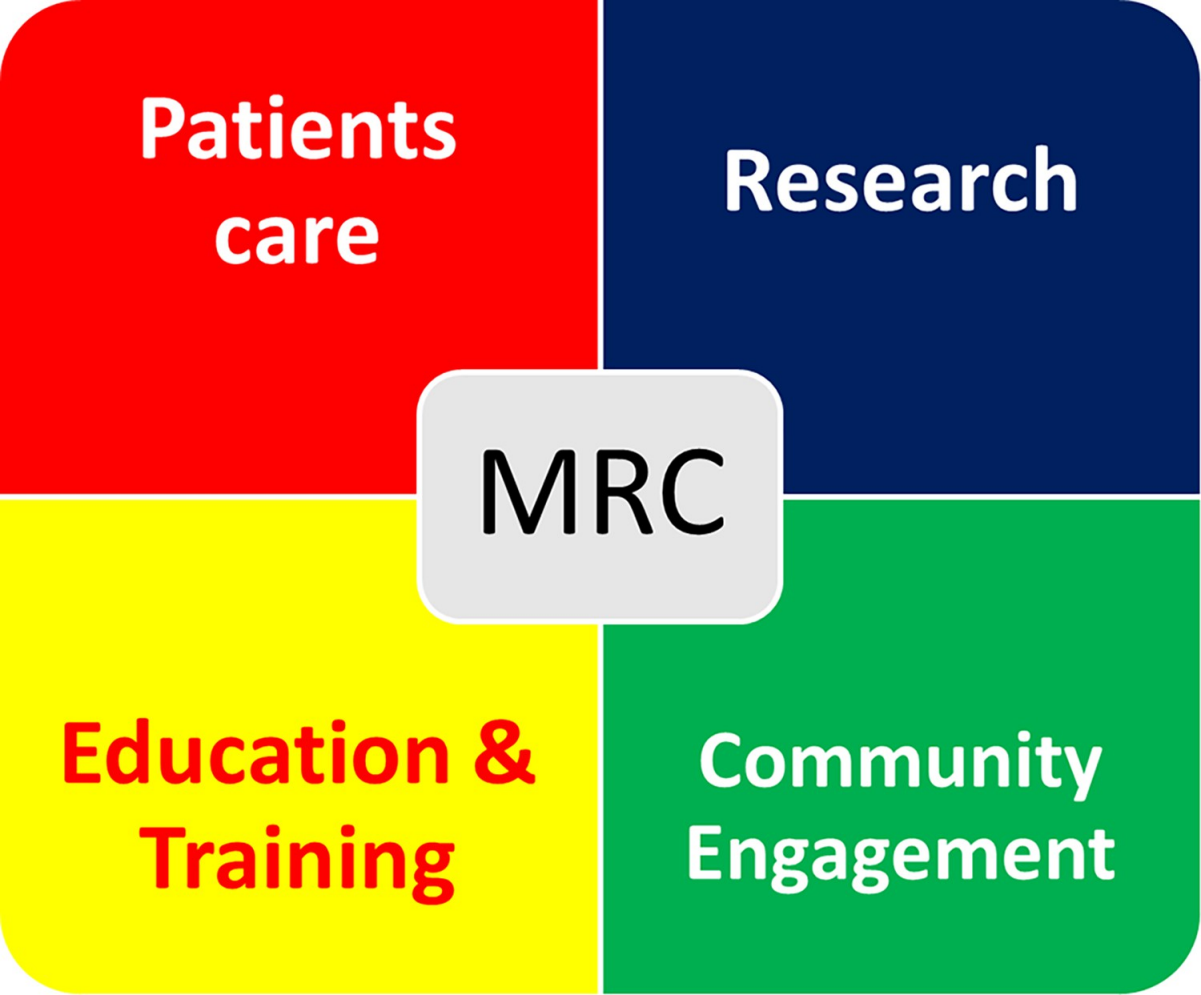
Mycetoma Research Center objectives align with the United Nation Sustainable Development Goals.

Apart from its core objective of patient care and discovering effective diagnostics and treatments for mycetoma, the MRC is deeply involved in diverse activities that align with the United Nations SDGs, illustrating its dedication to broader societal welfare. In this communication, we aim to share our experiences in these initiatives for mutual benefit and knowledge exchange on a global scale (Fig 1).

## Promoting health and well-being (SDGs 3,4,17)

The brunt of mycetoma burden falls disproportionately on those residing in rural areas, far removed from the reach of modern healthcare infrastructure. Geographical remoteness, inadequate facilities, and socioeconomic challenges form formidable barriers, hindering access to timely diagnosis and treatment. In these remote areas, the struggle against mycetoma is not just a battle against disease but also against social injustices [20].

MRC adopts a proactive stance, working in the heart of these communities. Understanding that traditional healthcare models may fall short in reaching those most in need, they adapt their approach, employing innovative strategies to bridge the gap [14]. The MRC has established several satellite centers in endemic regions to offer the affected population healthcare locally in remote villages. These centers are Wad Onsa in Sennar State, located 400 km from Khartoum, and Euaawa village in White Nile State, 350 km from Khartoum; over 1,000 patients with mycetoma have received comprehensive care here. Care at satellite centers include medical and surgical treatments, as well as diagnostic investigations such as ultrasound, x-ray, and histopathological examinations. In addition to medical care, health education sessions and socioeconomic support have been provided, all free of charge. Extensive fieldwork (>20 field missions) lies at the heart of the MRC’s outreach work, thus, it established teams of dedicated healthcare professionals to visit remote villages in underserved regions at Sennar, White Nile, and El Gazeria States and is equipped with medical supplies, diagnostic tools, and educational materials [12].

These field missions serve multiple purposes; they provide much-needed medical care to individuals suffering from mycetoma, raise awareness about the disease and its prevention, and establish crucial connections with local communities.

In addition to providing direct medical care, the MRC places a strong emphasis on education and capacity-strengthening initiatives. More than 700 health care providers in endemic regions have been trained on mycetoma diagnosis, treatment, and prevention strategies, empowering them to take ownership of healthcare delivery within their communities. Through workshops, seminars, and hands-on training sessions, the MRC equips healthcare workers with the knowledge and skills needed to effectively manage mycetoma cases, thereby reducing the burden of the disease at the grassroots level.

Recognizing the stark disparities in healthcare access and quality, particularly among marginalized communities, the MRC is committed to bridging these gaps and ensuring that every individual, regardless of their socioeconomic status or geographic location, has access to essential healthcare services. By meeting these challenges at its origin, the MRC provides a model of hope amidst the darkness, offering a glimmer of relief to those without a voice.

## Promoting community improvement (SDGs 1–17)

Community engagement is a cornerstone of the MRC’s approach. Rather than imposing external solutions, the center actively involves community leaders and members in decision-making processes, ensuring that interventions are culturally sensitive, sustainable, and tailored to local needs. By fostering trust and collaboration, the MRC builds enduring relationships with communities, laying the foundation for sustainable health improvements [12].

The MRC works closely with policymakers and healthcare authorities to advocate for improved healthcare infrastructure and policies that prioritize the needs of marginalized communities. By amplifying the voices of those often overlooked in decision-making processes, the center drives systemic, including policy change and fosters a more inclusive healthcare system.

By empowering communities and strengthening healthcare systems, the MRC brings us one step closer to achieving the vision of universal health coverage and the various SDGs (1–17).

## Promoting quality education and knowledge sharing (SDGs 4,17)

Recognizing that education is a powerful tool for empowerment and progress, the MRC goes beyond its primary research mandate to serve as a dynamic educational hub, nurturing the next generation of healthcare professionals, researchers, and students [21–25].

The MRC’s commitment to education begins with its research activities. As the center conducts groundbreaking research to understand mycetoma better, identify effective treatments, and develop innovative diagnostic tools, it simultaneously creates a fertile learning environment for students and aspiring researchers. Undergraduate and postgraduate students are welcomed, providing them with opportunities to engage in cutting-edge research projects, gain hands-on experience, and contribute to scientific advancements. This has resulted in >300 publications in prestigious scientific journals authored by a cohort of 30 graduate and postgraduate students.

Moreover, the MRC collaborates nationally and internationally with universities and research institutes to offer specialized training programmes in mycetoma diagnosis, treatment, and research methodologies. The MRC has organized 6 international conferences and seminars on mycetoma, and staff have presented at 200 different international meetings. These collaborative programmes provide students and healthcare professionals with access to world-class training resources, expert faculty members, and state-of-the-art facilities, fostering a culture of excellence and innovation in mycetoma research and care. Importantly, the MRC’s educational initiatives extend beyond theoretical knowledge to practical skills development. Through mentorship programmes, internships, and fieldwork opportunities, students and trainees are exposed to real-world challenges and solutions, preparing them to navigate complex healthcare landscapes and make meaningful contributions to public health practice.

Furthermore, the MRC places a strong emphasis on knowledge exchange and interdisciplinary collaboration. By bringing together experts from diverse backgrounds, including medicine, microbiology, epidemiology, and social sciences, the center fosters the exchange of ideas and perspectives, enriching the learning experience and driving innovation. Through seminars, workshops, and conferences, the MRC creates platforms for dialogue and debate, facilitating the exchange of best practices and emerging research findings.

By investing in education, the MRC not only builds a skilled workforce capable of addressing the complexities of mycetoma but also lays the groundwork for tackling other pressing public health challenges facing Sudan and beyond. The MRC’s educational initiatives exemplify the transformative potential of education in driving positive change and achieving SDGs.

## Promoting hygiene and environmental conditions (SDGs 3,6,12,13,14,15,16,17)

The microorganisms responsible for mycetoma thrive in the environment, finding conducive areas for their growth and spread in various substrates such as animal dung, soil particles, thorns, and foreign objects [26]. These diverse environmental niches provide the optimal conditions necessary for their growth and the spread of infection. Within these substrates, the microorganisms undergo developmental stages crucial for their pathogenicity, utilizing organic matter and environmental factors to proliferate and establish infection. This ecological interaction highlights the intricate relationship between the mycetoma-causing agents and their environment, emphasizing the significance of understanding these dynamics in combating the disease.

Aligned with its community infrastructure development initiatives, the MRC places emphasis on enhancing hygiene standards and promoting education and behavioral changes within communities. Collaborating closely with local health authorities, community leaders, and activists, the center conducts community-led workshops, training sessions, and outreach programs aimed at raising awareness about essential hygiene practices. These efforts encompass educating community members on the significance of wearing footwear to prevent injuries and infections, avoiding contact with dirt, thorns, and sharp objects, promoting regular handwashing, ensuring proper sanitation and animal husbandry facilities, and advocating for safe water management practices.

By equipping individuals with knowledge and practical skills, the MRC cultivates a culture of hygiene consciousness, fostering sustainable health behaviors that extend beyond addressing mycetoma. Moreover, the center encourages active participation from local villages in community cleaning initiatives, advocating the removal and proper disposal of waste and debris to reduce environmental contamination. Additionally, promoting reduced contact with animals and their dung (e.g., through redesigning dwellings) is emphasized as a preventive measure against mycetoma transmission. Through these comprehensive efforts, the MRC events empower communities to proactively safeguard their health and well-being while contributing to broader public health and environmental goals.

Furthermore, the MRC has pioneered the construction of 72 modern animal enclosures situated outside villages and in dwelling areas. This strategic measure aims to minimize human–animal contact, thereby mitigating the risk of disease transmission, particularly mycetoma, tickborne diseases, and other pathogens. In collaboration with an engineering company, this initiative exemplifies the MRC’s commitment to social responsibility and innovative problem-solving. By leveraging a public–private partnership model, the MRC ensures the implementation of sustainable solutions that address both public health concerns and environmental considerations. This collaborative effort not only enhances community well-being by reducing disease prevalence but also promotes coexistence between humans and animals while safeguarding the surrounding ecosystems.

## Fostering partnerships for global health (SDGs 3,17)

Fostering partnerships for global health is a cornerstone of the MRC approach to addressing mycetoma and advancing the broader agenda of sustainable development. Recognizing the complex and multifaceted nature of global health challenges, the MRC understands that collaboration and partnerships are essential for maximizing impact and achieving meaningful change.

The MRC recognizes that no single entity or organization can address mycetoma and other public health challenges in isolation. Therefore, the center actively engages with a diverse range of stakeholders, including government agencies, academic institutions, international organizations, and affected communities, to pool resources, expertise, and networks.

One of the key strategies employed by the MRC is to forge strategic partnerships with government agencies and NGOs at the local, national, and international levels. By aligning its goals and objectives with government priorities, the MRC ensures that its interventions are integrated into broader health policies and strategies. Moreover, partnering with government agencies enhances the sustainability and scalability of MRC initiatives, as they are embedded within existing health systems and structures. The MRC is actively collaborating with 15 national and 25 international global health centers, institutes, and NGOs to promote the United Nations SDGs.

Academic institutions play a crucial role in the MRC collaborative efforts, serving as hubs for research, innovation, and knowledge exchange. Through partnerships with universities and research institutions, the MRC advances understanding of mycetoma and develops novel approaches to prevention, diagnosis, and treatment. Collaborative research projects not only generate new knowledge but also build the capacity of researchers and strengthen research infrastructure in mycetoma-endemic regions.

International organizations provide valuable support and expertise in addressing global health challenges, including mycetoma. The MRC collaborates with organizations such as the WHO, the US Centers for Disease Control and Prevention (CDC), and non-governmental organizations (NGOs) such as the Drugs for Neglected Diseases initiative to coordinate efforts, share best practices, and advocate for increased attention and resources for mycetoma. By tapping into international networks and platforms, the MRC amplifies its voice and influence on the global stage, driving momentum for action and investment in mycetoma research and control.

Equally important are partnerships with affected communities, whose insights, experiences, and perspectives are invaluable in shaping effective and contextually appropriate interventions. The MRC adopts a participatory approach, engaging community members as active partners in the design, implementation, and evaluation of its programmes. By fostering trust, transparency, and mutual respect, the center builds strong, resilient partnerships that empower communities to take ownership of their health and well-being. By harnessing the collective expertise, resources, and networks of diverse stakeholders, the center maximizes its impact and contributes to global efforts to achieve health equity, sustainable development, and social justice. In so doing, the MRC exemplifies the transformative power of partnerships in driving positive change and improving health for all.

## Music and fine arts utility improving mycetoma awareness and advocacy (SDGs,3,4,11,16)

Harnessing the power of culture and creativity, the MRC pioneers a novel method for enhancing health awareness and advocacy. Recognizing the detrimental impact of late mycetoma diagnosis, the MRC has appointed many celebrities as Mycetoma Awareness and Advocacy Ambassadors. Under their leadership, the “We Are With You” group [27], comprising prominent Sudanese artists and media personalities, was established and embarked on a multifaceted advocacy journey. Through poetry, music, and public events, they raise awareness and educate communities across Sudan, culminating in a groundbreaking campaign and festival in Sennar State, Sudan, which is a highly endemic state for mycetoma. Embracing the fusion of fine arts with medical sciences, the MRC hosts a visual documentation workshop, uniting artists, educators, and policymakers to amplify mycetoma awareness. Through practical sessions and exhibitions, the workshop sparks dialogue and creativity, illustrating the transformative potential of art in health communication. Together, these initiatives mark a pioneering effort in mycetoma advocacy, fostering societal awareness, and engagement through the power of culture and creativity.

## Comprehending mycetoma: A disease linked to poverty (SDGs 1,2,3,4,5,8,10,16)

Mycetoma presents as a chronic and incapacitating condition. Its prevalence is notably high among individuals residing in impoverished rural regions, where access to adequate healthcare remains scarce. Apart from its physical symptoms, mycetoma imposes significant socioeconomic burdens on affected communities, frequently resulting in disability, social ostracization, and diminished productivity [11,12].

The MRC adopts a proactive approach to improve the quality of life of the affected patients, their families, and the community. The creation of the Mycetoma Vocational and Entrepreneurship Training Center, known as SAAI’D, represents a significant advancement in the MRC’s efforts to address the multifaceted challenges faced by individuals affected by mycetoma and disabilities. SAAI’D serves as a crucial institution dedicated to vocational training, entrepreneurship development, and community engagement, with a vision to foster equality, eliminate stigma, and empower individuals through skill enhancement and economic opportunities. It has equipped 250 participants with the necessary tools and knowledge for self-reliance and success while a dedicated board ensures governance, accountability, and transparency. Operating within eco-friendly facilities and supported by strategic partnerships, SAAI’D embody sustainability and environmental stewardship, with its establishment made possible through generous donations, reflecting a collective commitment to improving the well-being and empowerment of those affected by mycetoma and disabilities [28].

## Conclusion

In conclusion, mycetoma, a chronic and neglected tropical disease, poses substantial medical, health and socioeconomic challenges, particularly in impoverished rural areas with limited access to healthcare. Beyond its physical effects, mycetoma inflicts profound burdens on affected communities, including disability, social stigma, and reduced productivity. Recognizing these challenges, the MRC addresses the multifaceted dimensions of the disease. Through research, education, training, community engagement, and partnerships, the MRC not only combats mycetoma but also contributes to broader societal goals, exemplifying sustainable development principles. By understanding the unique needs of affected populations, implementing comprehensive strategies, and addressing the UN SDGs, the MRC provides a framework to fight against mycetoma and a model for combatting poverty-related health disparities.

## Disclaimer

The findings and conclusions in this report are those of the authors and do not necessarily represent the official position of the US Centers for Disease Control and Prevention.
